# Carotenoids from *Mangifera Pajang* and Their Antioxidant Capacity 

**DOI:** 10.3390/molecules15106699

**Published:** 2010-09-28

**Authors:** Hock-Eng Khoo, K. Nagendra Prasad, Amin Ismail, Nohaizan Mohd-Esa

**Affiliations:** 1 Department of Nutrition and Dietetics, Faculty of Medicine and Health Sciences, Universiti Putra Malaysia, 43400 UPM Serdang, Selangor, Malaysia; E-Mails: hockeng_khoo@yahoo.com (H.-E.K.); knag76@gmail.com (K.N.P.); nhaizan@medic.upm.edu.my (N.M.-E.); 2 Laboratory of Analysis and Authentication, Halal Products Research Institute, Universiti Putra, Malaysia, 43400 Serdang, Selangor, Malaysia

**Keywords:** antioxidant activity, bambangan, biological assay, carotenoids, DPPH

## Abstract

This study provides new data on the various carotenoids found in bambangan (*Mangifera pajang* Kosterm.) peel and pulp extracts, such as all-*trans*-α- and β-carotene, *cis*-β-carotene, 9-*cis*-β-carotene, and cryptoxanthin. Chemical and biological antioxidant assays were determined to evaluate the antioxidant capacity of bambangan peel and pulp extracts. Bambangan pulp had higher α- and β-carotene contents (7.96 ± 1.53 and 20.04 ± 1.01 mg/100 g) than its peel (4.2 ± 0.14 and 13.09 ± 0.28 mg/100 g); the cryptoxanthin contents of bambangan peel and pulp were 0.60 and 1.18 mg/100 g, respectively. The antioxidant activity results determined by chemical assay using the 2,2-diphenyl-2-picrylhydrazyl (DPPH) method showed that bambangan peel extract had higher DPPH radical scavenging activity than its pulp. In the biological assays bambangan peel and pulp had protective effects against hemoglobin and LDL oxidation at an extract concentration of 1 ppm. Bambangan peel is a therefore a potential source of natural antioxidants and could be utilized as a functional ingredient.

## 1. Introduction

Bambangan (*Mangifera pajang* Kosterm.) is an indigenous fruit of the Borneo region. The tree can grow up to 20 m in height and bears up to hundreds of semi-oval shaped fruits which are brown colored and can weigh up to 2 kg. The pulp of bambangan fruit represents 50–67% of the fruit’s total weight, is fibrous and juicy, with a specific aromatic flavor and strong smell, and can be eaten fresh. Bambangan peel is a major by-product of its pulp and juice processing industries. It is fibrous and may contain various bioactive compounds such as polyphenols and carotenoids, like those found in mango peel [1]. In Borneo, bambangan peel is commonly used for cooking curry.

**Figure 1:**
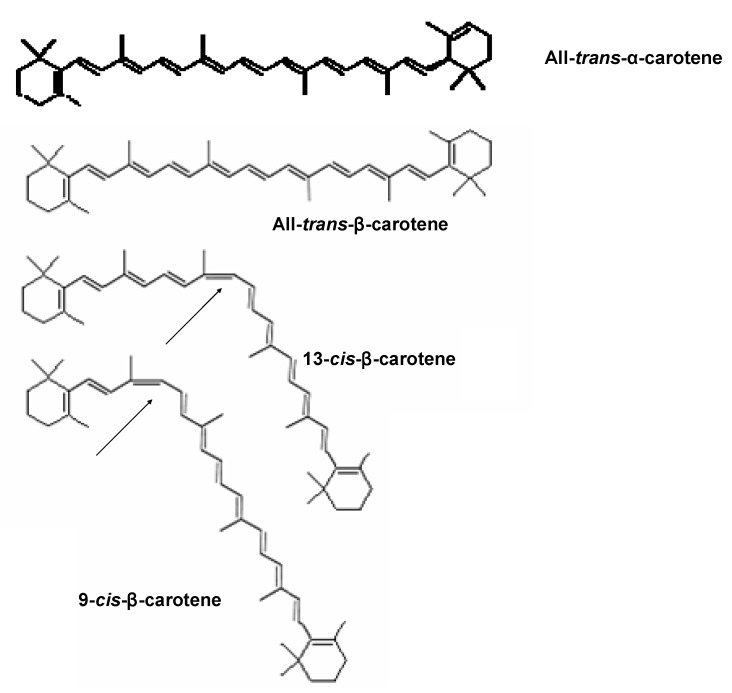
The structure of all-*trans*-α- and β-carotene and two other geometrical isomersof β-carotene. Source: ESA [5].

Among the various types of carotenoids, α- and β-carotene are commonly found in fruits. Carotenes are terpenes with the general formula C_40_H_x_ and have an unsaturated and long aliphatic hydrocarbon chain. Yellow-orange colored carotene occurs in several isomeric forms, such as α-, β-, γ‑, δ-, ε-, or ζ-carotene. The most common types of carotenoid are α- and β-carotene, which have several geometrical isomer forms. Isomers of β-carotene such as all-*trans*-β-carotene, 9-*cis*-β-carotene and 13-*cis*-β-carotene are commonly found in foods (Figure 1). Lutein and zeaxanthin are typical carotenoids abundantly found in vegetables. However, fruits have low concentration of lutein and zeaxanthin. Carotenoids are potential antioxidants and free radical scavengers. A previous study reported that carotenoids had protective effects against oxidation of liposomes [2]. Moreover, many researchers have argued that β-carotene had acceptable degree of antioxidant capacity and cardio-protective effect [3,4].

The amount of research on bambangan fruit has increased rapidly over the past few years,. Recent evidence suggests that bambangan fruit has acceptable amounts of β-carotene [6]. Previously, we have determined the isoflavone contents of bambangan fruit [7], while Abu Bakar *et al.* [8] have reported on its antioxidant activity, but little attention has been paid to the different carotenoids of this fruit. Therefore, the aim of this study is to determine and compare the carotenoid contents and isomers and the antioxidant capacity of bambangan peel and pulp extracts.

## 2. Results

### 2.1. High Performance Liquid Chromatography (HPLC) Analysis of Carotenoids in Bambangan Peel and Pulp Extracts

HPLC analysis was performed to determine the carotenoid contents in bambangan peel and pulp extracts. As a C_18_ column is not appropriate for determination of *cis*-isomers, therefore a C_30_ column was used for separation of these β-carotene isomers [9]. The percentages of recovery for α- and β-carotene were in the range of 80−105%. The LOD values for α- and β-carotene were 1.39 and 5.46 ppm, while LOQ values were 6.26 and 24.55 ppm, respectively. With the exception of several unresolved peaks a total of eight carotenoids were effectively resolved in the chromatograms of bambangan peel and pulp extracts. The separation factor (*α*) values for all peaks were greater than 1 (Table 1 and Table 2), which indicate a good selectivity of mobile phase to carotenoid composition.

**Table 1:** Retention time (min), factor (*k'*), separation factor (*α*), peak purity (%) and other identification parameters for geometry isomers of carotenoids in bambangan peel extract.

Peak no.	Carotenoid	Retention time	*k'*	*α*	Peak purity	Concentration*	*λ*_max_ (nm)		
1	Unknown	7.27 ± 0.3	3.33	1.38 (1,2)	93.5	−	398	420	440
2	Unknown	9.39 ± 0.2	4.59	1.41 (2,4)	99.0	−	424	444	470
3	−	−	−	−	−	−	−	−	−
4	Unknown	12.59 ± 0.3	6.49	1.84 (4,5)	99.7	−	402	425	446
5	Unknown	21.72 ± 0.1	11.93	1.15 (5,6)	99.0	−	402	428	446
6	Unknown	24.63 ± 0.2	13.66	1.08 (6,7)	99.9	−	404	428	450
7	Unknown	26.55 ± 0.3	14.80	1.06 (7,8)	99.5	−	404	428	450
8	Cryptoxanthin	28.10 ± 0.4	15.73	1.03 (8,9)	91.6	0.60 ± 0.001	424	444	472
9	*Cis*-cryptoxanthin	28.81 ± 0.1	16.15	1.09 (9,10)	99.0	0.07 ± 0.001	424	446	470
10	*Cis*-β-carotene	31.35 ± 0.3	17.66	1.24 (10,11)	96.6	2.87 ± 0.52	424	446	472
11	All-*trans*-α-carotene	38.53 ± 0.2	21.93	1.12 (11,12)	99.0	4.20 ± 0.14	428	450	476
12	*Cis*-β-carotene	42.97 ± 0.1	24.58	1.04 (12,13)	99.0	3.64 ± 0.48	428	453	476
13	All-*trans*-β-carotene	44.49 ± 0.1	25.48	1.09 (13,14)	99.9	13.09 ± 0.28	428	453	478
14	Unknown	48.36 ± 0.2	27.79	1.04 (14,15)	99.9	−	404	458	478
15	9-*Cis*-β-carotene	50.11 ± 0.2	28.83	1.04 (14,15)	99.4	2.53 ± 0.66	428	444	468

* The concentration of individual carotenoid is expressed as mean ± SD in mg/100 g.

Moreover, the retention factor (*k'*) values for all peaks ranged from 3.21 to 28.83, which also suggests that a suitable solvent strength was maintained. In addition, the purity factor for all peaks, except for peak number 4, was higher than 90% (Table 2). All peaks were identified based on the comparison of the retention time and absorption spectra between the studied samples and carotenoid standards. The *Q*-ratio was calculated for further confirmation of β-carotene *cis*-isomers, which was in the range of 0.1−0.23.

**Table 2:** Retention time (min), factor (*k'*), separation factor (*α*), peak purity (%) and other identification parameters for geometry isomers of carotenoids in bambangan pulp extract.

Peak no.	Carotenoid	Retention time	*k'*	*α*	Peak purity	Concentration*	*λ*_max_ (nm)		
1	Unknown	7.32 ± 0.2	3.21	1.36 (1,2)	96.3	−	400	402	444
2	Unknown	9.34 ± 0.3	4.37	1.17 (2,3)	99.0	−	416	442	468
3	Ζeta-carotene	11.72 ± 0.3	5.13	1.23(3,4)	99.0	−	398	422	444
4	Unknown	12.68 ± 0.2	6.29	2.09 (4,6)	85.2	−	400	422	446
5	−	−	−	−	−	−	−	−	−
6	Unknown	24.62 ± 0.1	13.15	1.09 (6,7)	99.6	−	424	444	464
7	Unknown	26.75 ± 0.3	14.37	1.06 (7,8)	99.9	−	424	444	464
8	Cryptoxanthin	28.23 ± 0.2	15.22	1.12 (8,10)	99.7	1.18 ± 0.01	424	444	472
9	−	−	−	−	−	−	−	−	−
10	*Cis*-β-carotene	31.51 ± 0.2	17.11	1.24 (10,11)	99.9	3.04 ± 0.13	424	444	470
11	All-*trans*-α-carotene	38.79 ± 0.2	21.29	1.07 (11,12)	99.5	7.96 ± 1.53	424	450	470
12	*Cis*-β-carotene	41.43 ± 0.3	22.81	1.08 (12,13)	99.0	3.74 ± 0.37	420	444	474
13	All-*trans*-β-carotene	44.69 ± 0.1	24.68	1.09 (13,14)	99.9	20.04 ± 1.01	428	453	478
14	Unknown	48.62 ± 0.3	26.94	1.05 (14,15)	99.0	−	402	453	487
15	9-*Cis*-β-carotene	50.90 ± 0.2	28.25	1.05 (14,15)	99.0	2.72 ± 0.10	426	444	472

* The concentration of individual carotenoid is expressed as mean ± SD in mg/100 g.

**Figure 2:**
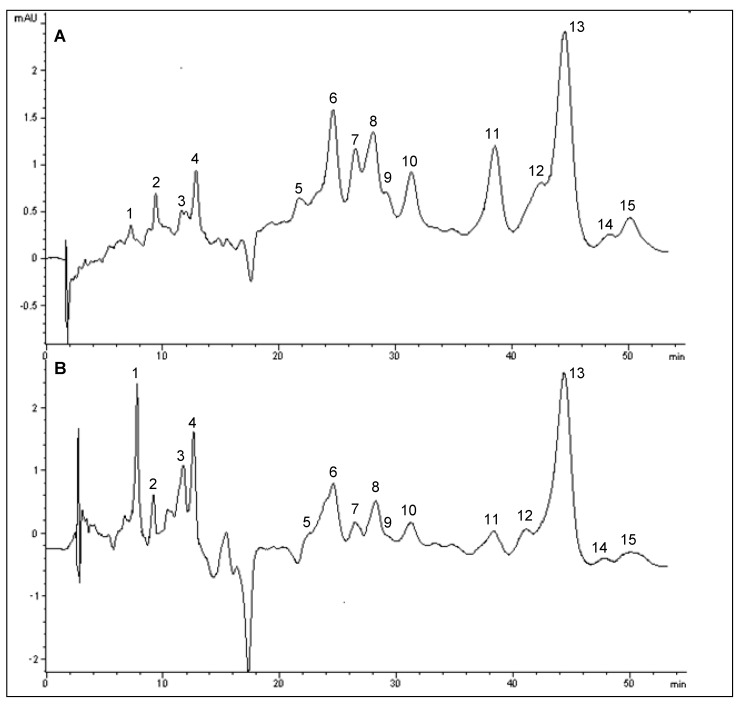
HPLC chromatogram of carotenoids in bambangan peel (A) and pulp (B) extracts.

In this study, a total of 15 peaks were found in bambangan peel extract (Figure 2A). Peaks 8 and 9 were identified as cryptoxanthin and *cis*-cryptoxanthin, peaks 10 and 12 were identified as *cis-*β-carotene, while peaks 11−13 were identified as α- and β-carotene, as further confirmed by spiking with α- and β-carotene standards. Moreover, peak 15 was identified as 9-*cis*-β-carotene, based on its bsorption spectra. The chromatogram profile of bambangan pulp was similar to that of peel (Figure 2), except for the zeta-carotene (peak 3) in bambangan pulp. *cis*-Cryptoxanthin was not detected in pulp along with other unidentified peaks. Additionally, the peaks of zeta-carotene and *cis*-cryptoxanthin were tentatively identified based on the absorption spectra. The UV-visible spectra for the identified carotenoids are in agreement with Rodriguez-Amaya and Kimura [10] The peaks of cryptoxanthin, α- and β-carotene were quantified by standard calibrations based on peak area, while all identified *cis*-β-carotenes were quantified using β-carotene standard calibration curve.

The results of this study showed that bambangan peel extract had 4.2 ± 0.14 and 13.09 ± 0.28 mg/100 g of α- and β-carotene (Table 1), while pulp contained 7.96 ± 1.53 and 20.04 ± 1.01 mg/100 g of α- and β-carotene respectively (Table 2). Therefore, α- and β-carotene contents of bambangan peel extract were significantly lower than pulp (*p* < 0.05). For bambangan peel and pulp extracts, 2.53 and 2.72 mg/100 g of 9-*cis*-β-carotene were found. Besides, other *cis*-isomers of β-carotene were identified in peel and pulp extracts ranged from 2.87–3.74 mg/100 g of fruit.

### 2.2. Antioxidant Activity of Chemical Assay

Due to low solubility of carotenoids in hexane, low concentrations (0.1–2.0 ppm of β-carotene equivalents) of carotenoid-rich sample extracts were used for the chemical and biological assays. A low concentration of carotenoid compound, especially β-carotene, was able to fully dissolve in absolute methanol [10,11,12]. By applying a semi-aqueous phase system, carotenoids were able to scavenge free radicals as antioxidant [13,14].

**Figure 3:**
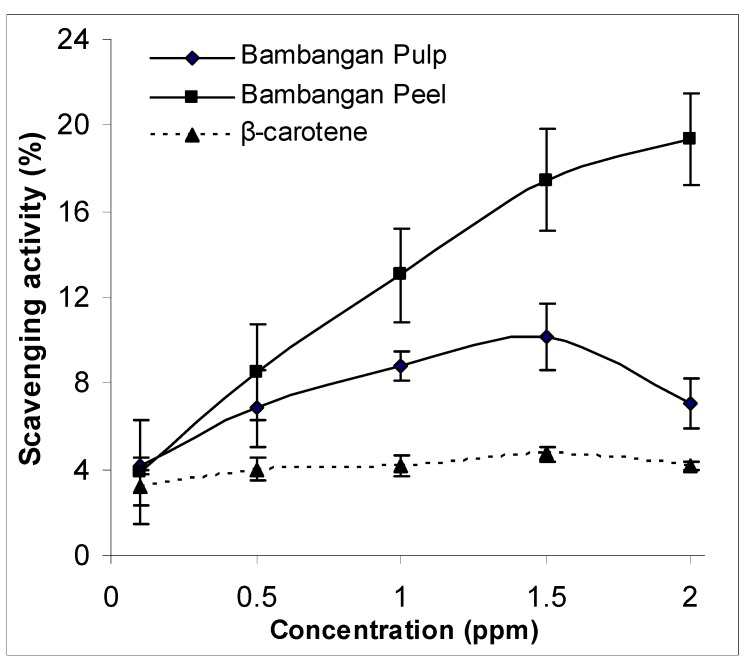
DPPH scavenging activity between bambangan peel and pulp extracts.

The scavenging activity (%) against DPPH radicals for bambangan peel and pulp extracts is shown in Figure 3. Results showed that the percentage scavenging activity of peel and pulp extracts at 0.1 ppm were not statistically different. However, a significant different was found between bambangan peel and pulp extracts at higher concentrations (0.5–2.0 ppm) (p < 0.05). The scavenging activity of the peel extract was concentration dependent. Compared to bambangan peel extract, the scavenging activity of pulp extract decreased when the concentration reaches an optimum level of 1.5 ppm. This finding is in agreement with Jimenez-Escrig *et al.* [11] that the scavenging effects of the superoxide anion free radical decreased with increasing extract concentration. In addition, the scavenging activity was significantly higher in bambangan peel and pulp extracts compared to β-carotene standard.

### 2.3. Antioxidant Activity of Biological Assays

The reliability of chemical assays for antioxidant determination, especially those involved in hydrogen transfer pathways, is questionable [15]. For this reason, biological assays were performed to determine the antioxidant activity of bambangan peels and pulp extracts. In this study, hemoglobin and LDL oxidation assays were chosen for determination as they are mimicking human biological system [16].

**Figure 4:**
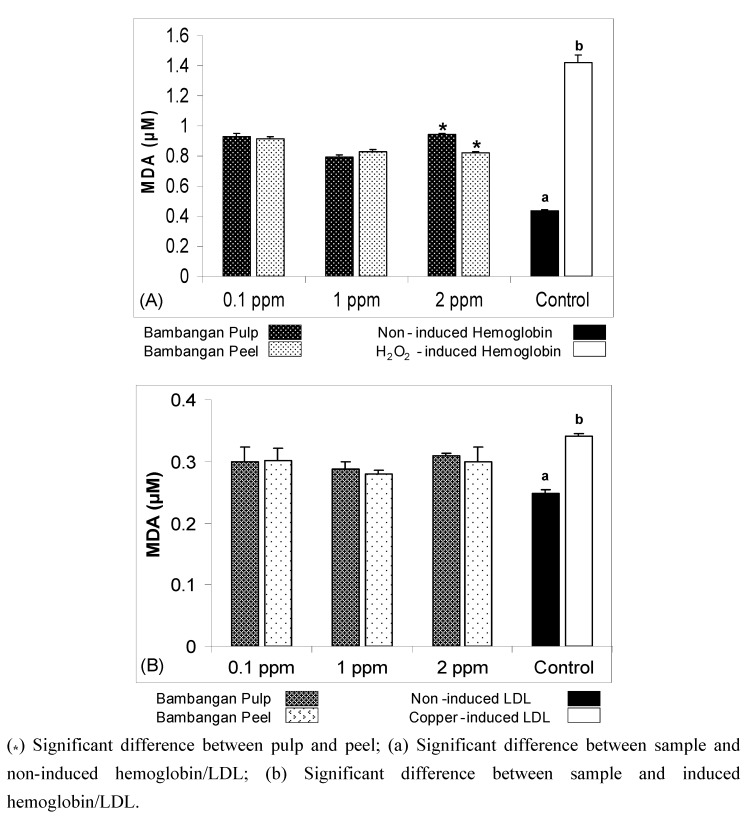
Comparison of malondialdehyde (MDA) equivalents between LDL oxidation treated with bambangan peels and pulp extracts (A); copper induced oxidation treated with bambangan peels and pulp extracts (B).

The result showed that compared to pulp bambangan peel extracts had protective effect against hemoglobin oxidation. MDA equivalents were used to express the biological assay results. The MDA equivalent of hemoglobin oxidation treated with bambangan peels extract was significantly lower (*p* < 0.05) than pulp extract at 2 ppm concentration (Figure 4A). The MDA equivalents of H_2_O_2_-induced hemoglobin oxidation treated with three different extract concentrations (0.1, 1 and 2 ppm) significantly decreased (*p* < 0.05) compared to H_2_O_2_-induced control. Therefore, both bambangan peels and pulp extracts had protective effect against H_2_O_2_-induced oxidation.

The results showed that both bambangan peels and pulp extracts had protective effect against copper-induced LDL oxidation using the three studied concentration (0.1, 1 and 2 ppm), in which MDA equivalent levels significantly reduced compared to copper-induced control (Figure 4B). The results suggest that bambangan peels and pulp extracts have protective effects against LDL oxidation at 1 ppm concentration. Moreover, the MDA equivalents did not show any significant differences between bambangan peels and pulp extract at three different concentrations. However, low extract concentration is best in the free radical scavenging model, where higher concentrations of carotenoid may exacerbate oxidation [17].

## 3. Discussion

The carotenoid contents of bambangan fruit found in this study were higher than those of other *Mangifera* fruits. The result was supported by Idris and Idris [6], who reported that *Mangifera indica* and *M. pentandra* fruits had 10 and 3 mg of β-carotene per 100 g (dry basis). The fruits of *M. foetida* contain 2.6–4.8 mg of total carotenoids per 100 g [18]. However, the carotenoid contents of the studied bambangan peel mainly consisted of carotenes and trace amounts of xanthophyll. Besides, bambangan peel and pulp extracts were confirmed to have carotenoids, such as α- and β-carotene, cryptoxanthin and carotenoid isomers, along with unidentified compounds. Moreover, α- and β-carotene contents of bambangan pulp were higher than its peel. In addition, the occurrence of cryptoxanthin in bambangan pulp is relatively new, compared to mango pulp [9].

The DPPH radical scavenging assay is one of the chemical assays involving an electron transfer pathway, and commonly used to determine the activity of antioxidant compounds. Apparently, this finding suggests that carotenoids have lower scavenging activity than most of the polar compounds measured using the DPPH assay. Another interesting finding was that the studied bambangan peel extract had a low carotenoid content, but it had high scavenging activity. If compared to bambangan pulp extract, it had higher carotenoid content; the fact suggests that β-carotene is a weaker antioxidant as compared to non-carotenoid compounds, which also has lower scavenging activity. Besides, bambangan peel extract had higher scavenging ability than β-carotene. As shown in the result, the scavenging activity decreased when the carotenoid-rich extract reaches an optimum level of 1.5 ppm. At low concentrations, carotenoids, especially β-carotene, have strong antioxidant effects. However, a high concentration of carotenoid in biological system has been reported to have no protective effect against LDL oxidation, since they might act as pro-oxidants [19].

There are several possible explanations for LDL oxidation being one of the major risk factors for cardiovascular disease. Lau [20] has discussed how natural antioxidants that help to suppress LDL oxidation, could also reduce the risk of cardiovascular disease; while Heinecke [21] has reported on lipoprotein oxidation in cardiovascular disease. However, our study found that moderate concentration of bambangan peel and pulp extracts have protective effect against copper-induced LDL oxidation. Thus, it can be suggested that the concentration of the bambangan extracts is an important aspect of this study. Furthermore, a high extract concentration will reduce the scavenging activity, while low amounts of bioactive substances may have minimal or no protective effects. This was supported by Li *et al.* [22] who claimed that a high dose of lotus germ oil has pro-oxidation effects. In the biological system, the studied bambangan peel extract was able to significantly reduce the secondary oxidation product, MDA, produced from H_2_O_2_-induced hemoglobin oxidation, as compared to its pulp extract. Ajila *et al.* [23] support our findings in that mango peel extract exhibited a high antioxidant activity. Our study was also in agreement with Ajila and Prasada Rao [1] who reported that mango peel extracts protected erythrocytes against oxidative stress.

In Nature, fruit peel has the ability to protect the pulp from various types of environmental changes, including heat and oxidative stresses that might due to chemical and biological reactions. A laboratory test carried out by Sapitnitskaya *et al.* [24] showed that grapefruit peel tissues allow the fruit to respond adequately to chilling. Similarly, antioxidant substances found in bambangan peel enable to protect the pulp against environmental changes. These substances could be other types of carotenoids and polyphenolic compounds. Besides, the carotenoid content of the studied bambangan peel was lower than its pulp. The possible substances that might contribute to the protective effect could be xanthophyll and polyphenolic compounds, which were not determined. Moreover, Gil *et al.* [25] reported that the antioxidant activities of phenolic compounds were greater than those of carotenoids. A future study with more focus on polyphenolics and antioxidant capacity of bambangan peel is needed, which should also include various extraction methods.

## 4. Materials and Methods

### 4.1. Chemicals and Standards

Carotene standards (α- and β-carotene, HPLC grade), bovine serum albumin (BSA), 2,2-diphenyl-2-picrylhydrazyl (DPPH), 60% iodixanol (OptiPrep), trichloroacetic acid and thiobarbituric acid (for TBA assay), sodium azide, Tris-HCl buffer, phosphate buffer tablet and Hepes were obtained from Sigma-Aldrich Co. (St. Louis, MO, USA); methanol (HPLC grade), dichloromethane (HPLC grade), isopropanol (HPLC grade) and *n*-hexane were purchased from Fisher Scientific (Waltham, MA, USA). Sodium chloride, sodium carbonate, sodium hydroxide, potassium sodium tartrate, copper (II) chloride (CuCl_2_), hexane (extraction solvent), Folin-Ciocalteu reagent (for protein determination) and hydrogen peroxide (H_2_O­_2_) were obtained from Merck (Darmstadt, Germany); while HCl for TBA assay was purchased from Scharlau (Barcelona, Spain) and ethylenediaminetetraacetic acid (EDTA) was obtained from Calbiochem (EMD Chemicals, NJ, USA).

### 4.2. Extraction of Sample

Fresh bambangan fruits and information on their commercial ripening stage were obtained from Sarawak, Malaysia. The fruit was manually peeled to separate it from the pulp, and its kernel was removed. Then the pulp and peel were stored at −80 °C before freeze drying using a bench top freeze dryer (Virtis, NY, USA). Lyophilized samples were ground into fine particles using a grinder and sieved (particle size of 20 mesh). The sample powders were stored at −20 °C before extraction. Lyophilized pulp and peel (1.0 g) were mixed with hexane (20 mL). The mixtures were stirred using a Unimax 1010 DT shaking incubator (Heidolph, Schwabach, Germany). After 5 min, the hexane layers were separated from the residue and re-extracted again with hexane until the layer become colorless. The pooled hexane fraction was separated from the residue by centrifugation at 1,000 g for 10 min. The hexane was evaporated from the extract using a Büchi rotary evaporator (Flawil, Switzerland). The resulting extracts were completely dried by purging with nitrogen gas and then stored at −80 °C in amber bottles. The extracts were re-dissolved in dichloromethane and absolute methanol for HPLC and antioxidant analysis, respectively.

### 4.3. HPLC Determination of Carotenoids

Identification and quantification of carotenoids in bambangan peel and pulp extracts were carried out using the HPLC method as described by Tai and Chen [26] with some modifications. HPLC analysis was carried out using a Hewlett Packard (HP1100) system (Agilent Technologies, Palo Alto, CA, USA), coupled with diode array detector. A 150 × 4.6 mm, 3 µm C_30_ analytical column (Waters Co., Milford, MA, USA) was used in this analysis. The mobile phase consisted of methanol- dichloromethane-isopropanol (89:1:10, *v*/*v*/*v*). The carotenoid separation was performed by isocratic elution at a flow rate of 1.0 mL/min. The injection volume was 20 μL, with the column temperature at 25 °C. The detection was carried out at absorption of 453 nm.

### 4.4. Identification of Carotenoids

The carotenoid contents of bambangan peel and pulp were identified using the HPLC system software by comparing the retention time (RT) and absorption spectra of unknown peaks with reference standards. In addition, identification was done by spiking carotenoid standards into the samples. Furthermore, the *cis*-isomers of carotenoids were identified based on the spectral characteristics and *Q*-ratio values reported by previous literature [9,10]. Quantification was done by external standard calibration based on the peak area.

### 4.5. Preparation of Standard Curve and Recovery

Several concentrations of all-*trans*-α- and β-carotene solution (3–80 ppm) were injected into HPLC, and the linear regression equation for each standard curve was obtained. The regression equation and correlation coefficient (*r^2^*) were calculated using Microsoft Excel, and the *r^2^* for all standards was more than 0.99. The limit of detection (LOD) and limit of quantification (LOQ) were measured based on a method described by Holcombe [27]. LOD and LOQ are referred to the smallest amount or concentration of a compound that can be estimated or quantified with acceptable reliability [28]. Three concentrations of α-carotene (2.9, 5.7, 20.3 ppm) and β-carotene (7.1, 22.2, 79.7 ppm) were prepared for the recovery tests.

### 4.6. Antioxidant Capacity

The antioxidant capacity of bambangan peel and pulp extracts were determined by chemical (2,2-diphenyl-2-picrylhydrazyl) and biological (hemoglobin and copper-induced oxidation) assays.

#### 4.6.1. 2,2-Diphenyl-2-picrylhydrazyl (DPPH) Radical Scavenging Assay

The scavenging activity of bambangan peel and pulp extracts was performed based on DPPH radical scavenging assay as described by Lai *et al.* [29] with some modifications. The analysis was carried out by addition of carotenoid-rich sample extract obtained from peel and pulp of bambangan (0.5–2.0 ppm of β-carotene equivalents dissolved in methanol) to 0.1 M Tris-HCl buffer (0.8 mL, pH 7.4) and 0.5 mM DPPH solution (1.0 mL). The mixture was well shaken and kept for 20 min at room temperature. The absorbance was read at 517 nm against a blank. The percentage scavenging activity was calculated using the following equation:

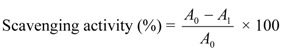
(1)
where *A*_0_ is the absorbance of the control, and *A*_1_ is the absorbance of the sample extract.

#### 4.6.2. RBC preparation and LDL Isolation

Fasting venous blood (10 mL) from healthy volunteers (aged 20–30 years) were collected in EDTA tubes (0.4 g/L). As described by Chu and Liu [30], the tube was centrifuged at 1,600 g for 20 min at 15 °C to separate red blood cells (RBC) from the blood. The RBC was washed with phosphate buffer saline (PBS) three times. LDL was isolated from prepared plasma by sequential ultracentrifugation at 16 °C at 33,000 g for 3 h using an Optima L-100 K ultracentrifuge (Beckman, Brea, CA, USA) with slow acceleration to 305 g. The procedure was based on a method developed by Graham *et al.* [31] with some modifications. Plasma (4 mL) was mixed with 60% iodixanol (1 mL) and 4 mL was transferred to Optiseal tube. The mixture was overlaid with 20% of iodixanol before the tube was filled up with Hepes-buffer saline. After ultracentrifugation, LDL-containing fractions were removed from the lipoprotein layer, which can be seen as orange-brownish colored layer. The protein content of LDL-containing fraction was determined by Lowry’s method [32] using bovine serum albumin as the standard.

#### 4.6.3. Copper-induced LDL Oxidation

Low density lipoprotein (LDL) oxidation was carried out based on a method described by Tsoukatos *et al.* [33] with some modifications. Pooled LDL-containing fractions were suspended in PBS (1.7 mL, pH 7.4) in a final volume of 2 mL containing 80 g/L LDL protein and 4 µmol/L CuCl_2_, and incubated at 37 °C for 3 h with and without addition of carotenoid-rich sample extracts (0.1, 1 and 2 ppm of of β-carotene equivalents in methanol, 0.1 mL). The control analysis was performed without addition of CuCl_2_ and sample extract. The oxidation was terminated by addition of EDTA (0.01%, final concentration). Malondialdehyde (MDA) produced from LDL oxidation was measured using TBA assay as described by Buege and Aust [34].

#### 4.6.4. Hemoglobin Oxidation Assay

Hemoglobin oxidation was performed using a method previously described by Rodríguez *et al.* [35] with slight modifications. The experiment was carried out within a day of blood withdrawal. The red blood cells (RBCs) were gently re-suspended with PBS to obtain 5% of RBC and pre-incubated at 37 °C for 10 min in the presence of 1 mmol/L sodium azide (to inhibit microbial growth). Subsequently, 1.6 mL of RBC was transferred to test tube for oxidation analysis. All test tubes except control tube were added with 10 mmol/L of H_2_O_2_ and with or without the addition of carotenoid-rich sample extract (0.1, 1 and 2 ppm of of β-carotene equivalents in methanol, 0.2 mL). After 60 min incubation at 37 °C, the mixture was kept for 60 sec in an ice bath and centrifuged at 1853*g* for 10 min at 4 °C. The MDA equivalents were measured using TBA assay as described before.

### 4.7. Statistical Analysis

Data were presented as mean ± standard deviation of three determinations. Data were subjected to independent sample t-test and analysis of variance (ANOVA). The percentage of scavenging activity was compared between the bambangan peel and pulp extracts, and with standard (β-carotene) for chemical assay. The mean values of MDA produced were compared between the sample extracts and control. The significant level was set at *p* < 0.05. SPSS for Windows version 15.0 was used for statistical analysis.

## 5. Conclusions

This study examined the carotenoid content and antioxidant capacity of bambangan peel and pulp. The result has shown that bambangan peel extract had an acceptable level of antioxidant capacity in the studied chemical and biological assays. The evidence from this study suggests that carotenoid-rich bambangan peel can be a potential functional food. Further investigations are needed to determine other polyphenolic compounds in bambangan peel and pulp, and their corresponding biological activity.
